# Nitric Oxide in Selective Cerebral Perfusion Could Enhance Neuroprotection During Aortic Arch Surgery

**DOI:** 10.3389/fcvm.2021.772065

**Published:** 2022-01-14

**Authors:** Daniele Linardi, Romel Mani, Angela Murari, Sissi Dolci, Loris Mannino, Ilaria Decimo, Maddalena Tessari, Sara Martinazzi, Leonardo Gottin, Giovanni B. Luciani, Giuseppe Faggian, Alessio Rungatscher

**Affiliations:** ^1^Division of Cardiac Surgery, Department of Surgery, University of Verona, Verona, Italy; ^2^Department of Pharmacology, University of Verona, Verona, Italy; ^3^Division of Cardio-Thoracic Anesthesiology and Intensive Care, Department of Surgery, University of Verona, Verona, Italy

**Keywords:** hypothermic circulatory arrest (HCA), nitric oxide (NO), selective cerebral perfusion, neuroprotection, aortic arch surgery/hemiarch repair

## Abstract

**Background:**

Hypothermic circulatory arrest (HCA) in aortic arch surgery has a significant risk of neurological injury despite the newest protective techniques and strategies. Nitric oxide (NO) could exert a protective role, reduce infarct area and increase cerebral perfusion. This study aims to investigate the possible neuroprotective effects of NO administered in the oxygenator of selective antegrade cerebral perfusion (SCP) during HCA.

**Methods:**

Thirty male SD adult rats (450–550 g) underwent cardiopulmonary bypass (CPB), cooling to 22°C body core temperature followed by 30 min of HCA. Rats were randomized to receive SCP or SCP added with NO (20 ppm) administered through the oxygenator (SCP-NO). All animals underwent CPB-assisted rewarming to a target temperature of 35°C in 60 min. At the end of the experiment, rats were sacrificed, and brain collected. Immunofluorescence analysis was performed in blind conditions.

**Results:**

Neuroinflammation assessed by allograft inflammatory factor 1 or ionized calcium-binding adapter molecule 1 expression, a microglia activation marker was lower in SCP-NO compared to SCP (4.11 ± 0.59 vs. 6.02 ± 0.18%; *p* < 0.05). Oxidative stress measured by 8oxodG, was reduced in SCP-NO (0.37 ± 0.01 vs. 1.03 ± 0.16%; *p* < 0.05). Brain hypoxic area extent, analyzed by thiols oxidation was attenuated in SCP-NO (1.85 ± 0.10 vs. 2.74 ± 0.19%; *p* < 0.05). Furthermore, the apoptotic marker caspases 3 was significantly reduced in SCP-NO (10.64 ± 0.37 vs. 12.61 ± 0.88%; *p* < 0.05).

**Conclusions:**

Nitric oxide administration in the oxygenator during SCP and HCA improves neuroprotection by decreasing neuroinflammation, optimizing oxygen delivery by reducing oxidative stress and hypoxic areas, finally decreasing apoptosis.

## Introduction

Hypothermic circulatory arrest (HCA) is required during aortic arch surgery (1). Since the 1990's, selective brain perfusion (SCP) techniques have become increasingly common (2, 3). Despite these advancements, neurological damage of different severity develops in 5–20% of patients, mainly in urgent interventions for aortic dissection (4).

Neurological damage comprises permanent neurological dysfunction (PND) and temporary neurological dysfunction (TND). These two types of damage differ for etiopathogenesis, histological and clinical manifestation. PND manifests itself clinically as a neurological deficit secondary to perfusion deficit (embolism, vascular occlusion, and absence of blood flow). Risk factors for PND include an atheromatous disease and vasculopathy of the epiaortic vessels and the cerebral circulation (5, 6). TND is a reversible and widespread lesion attributed primarily to a global brain ischemic lesion consequence of inadequate brain protection (7).

**Figure 1:**
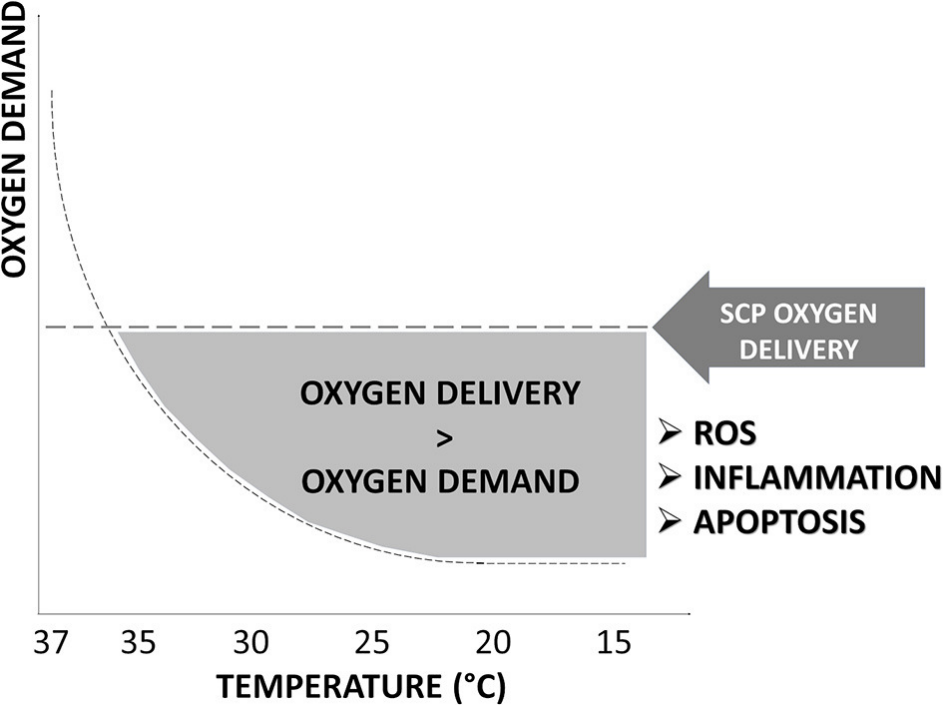
Oxygen delivery in hypothermia. The excess of oxygen delivered by selective cerebral perfusion (SCP) compared to decreased oxygen demand at the different grades of hypothermia is represented by the gray area under “SCP oxygen dysfunction.” An excess in oxygen delivery increases reactive oxygen species (ROS), oxidative stress, inflammation, and apoptosis and promotes temporary neurologic deficit (TND) development. SCP flow rate, oxygen content, temperature, and blood gas management of the perfusate have not been clearly defined and vary considerably among centers.

Although techniques advancements and developments have reduced PND, the percentage of patients with TND is still exceedingly high (8). A combination of hypothermia and SCP could expose the brain to an excess of oxygen delivery related to reduced demand during hypothermia (9). This could impair cerebral microvascular autoregulation with the production of reactive oxygen species (ROS), oxidative stress, and inflammation that could contribute to TND development (Figure 1).

Nitric oxide (NO) has been recognized for its cardioprotective and neuroprotective effects (10). Since constitutive NO synthase (cNOS) is impaired during hypothermia (11), NO administration could effectively optimize cerebral microvascular blood flow, attenuating ischemia-reperfusion injury, neuroinflammation, and neuronal death (12). This preclinical study aims to investigate the neuroprotective effects of NO administered through SCP in HCA.

## Methods

All experiments were performed according to the Guide for the Care and Use of Laboratory Animals (National Institutes of Health Publication No. 85-23, revised 1996). The experimental protocol was validated by the scientific committee for experimental research of the University of Verona and approved by the National Ministry of Health (Ministerial Authorization Number 568/2020-PR, Protocol Number 56DC9.54).

A total of 30 male Sprague Dawley rats weighing 450 ± 50 g, aged 6–7 months, were used for the experiments.

### Surgical Procedure

Anesthesia was induced with Sevoflurane 6% (Forene; Abbott, Baar, Switzerland), rats were orotracheal intubated and ventilated by mechanical respirator for rodents (Harvard Model 687, Harvard Apparatus, Holliston, MA, USA). Tidal volume was 10 ml/kg and respiratory frequency 70 breaths per minute, animals were kept sedated with a mixture of oxygen and sevoflurane 2%, ketoprofen 2 mg/kg was injected subcutaneously to maintain analgesia, and 2 mg/kg of pancuronium-bromide was infused through the right femoral vein to obtain muscle relaxation. Electrocardiogram and body temperature through a rectal probe were monitored throughout the entire experiment. Furthermore, a miniaturized 2-Fr-diameter catheter (model SPR 838, Millar Instruments, Houston, TX, USA) was inserted into the right femoral artery to monitor systemic arterial pressure.

Animals were surgically prepared for cardiopulmonary bypass (CPB). Rat CPB model was previously described in detail (13–15). The circuit was modified to permit selective cerebral perfusion. After right external jugular isolation, a 5-Fr-diameter cannula was inserted and advanced in the right atrium, and 500 IU/kg of heparin was administered. The left common carotid artery was cannulated with a 24 G catheter advanced to the aortic arch, and another 26 G catheter was inserted in the upper left carotid artery and directed to the brain. The two cannulas were connected through a three-way stopcock to the arterial perfusion line.

The extracorporeal circulation circuit was constituted by an outflow tube connected to the right jugular vein, a venous reservoir connected to vacuum (maintained between −40 and −60 cmH_2_O), a connection tube that runs through a roller pump (Stockert SIII, Sorin, Germany) and ends in a hollow fiber oxygenator with industrial standard characteristics (Sorin, Mirandola, MO, Italy), and an inflow tube connected through a three-way stopcock to upper and lower left carotid artery. The total length of the extracorporeal circuit was 90 cm, outflow tube had 2 mm inside diameter whereas the remaining circuit had 1.6 mm inside diameter; total filling volume was 6.5 ml and constituted by 50% lactated Ringer's solution and 50% colloid solution (Voluven, Fresenius Kabi Italia Srl, VR, Italy). The oxygenator exchange surface area was 450 cm^2^. A flow rate of 110–130 ml/kg/min and a range of mean arterial pressure of 70–80 mmHg was assured. The temperature was adjusted by a heat exchanger (Sarns cardioplegic set, Terumo Cardiovascular System Corp., Ann Arbor, MI, USA) incorporated in the circuit, and it was modulated upon rectal temperature monitoring.

### Experimental Protocol

Hypothermia (22°C) was induced in 25 min. CPB was conducted with the total assisted flow (110–130 ml/kg). When the temperature was 22 ± 1°C, the aorta was clamped, and rats were left in a state of HCA. Immediately the three-way stopcock on the arterial line was turned to direct the oxygenated blood flow only to the cannula inserted in the upper left carotid and, therefore, to the brain. In its gaseous form, NO could be administered to the patient through the CPB, connecting the NO delivery system directly to the oxygenator (16, 17).

Animals were randomized to receive unilateral SCP (10 ml/kg/min) with oxygenated blood (group SCP; *n* = 15) or with the addition of NO (20 ppm) using a gas delivery system (iNOmax, Mallinckrodt Staines-upon-Thames, United Kingdom) connected directly to the oxygenator (group SCP-NO; *n* = 15).

The experimental model has been evaluated in preliminary experiments to measure the blood flow through the cannula inserted in the superior left carotid artery, the cerebral electrical activity measured by continuous EEG, and methemoglobin production on right jugular vein blood samples.

**Figure 2:**
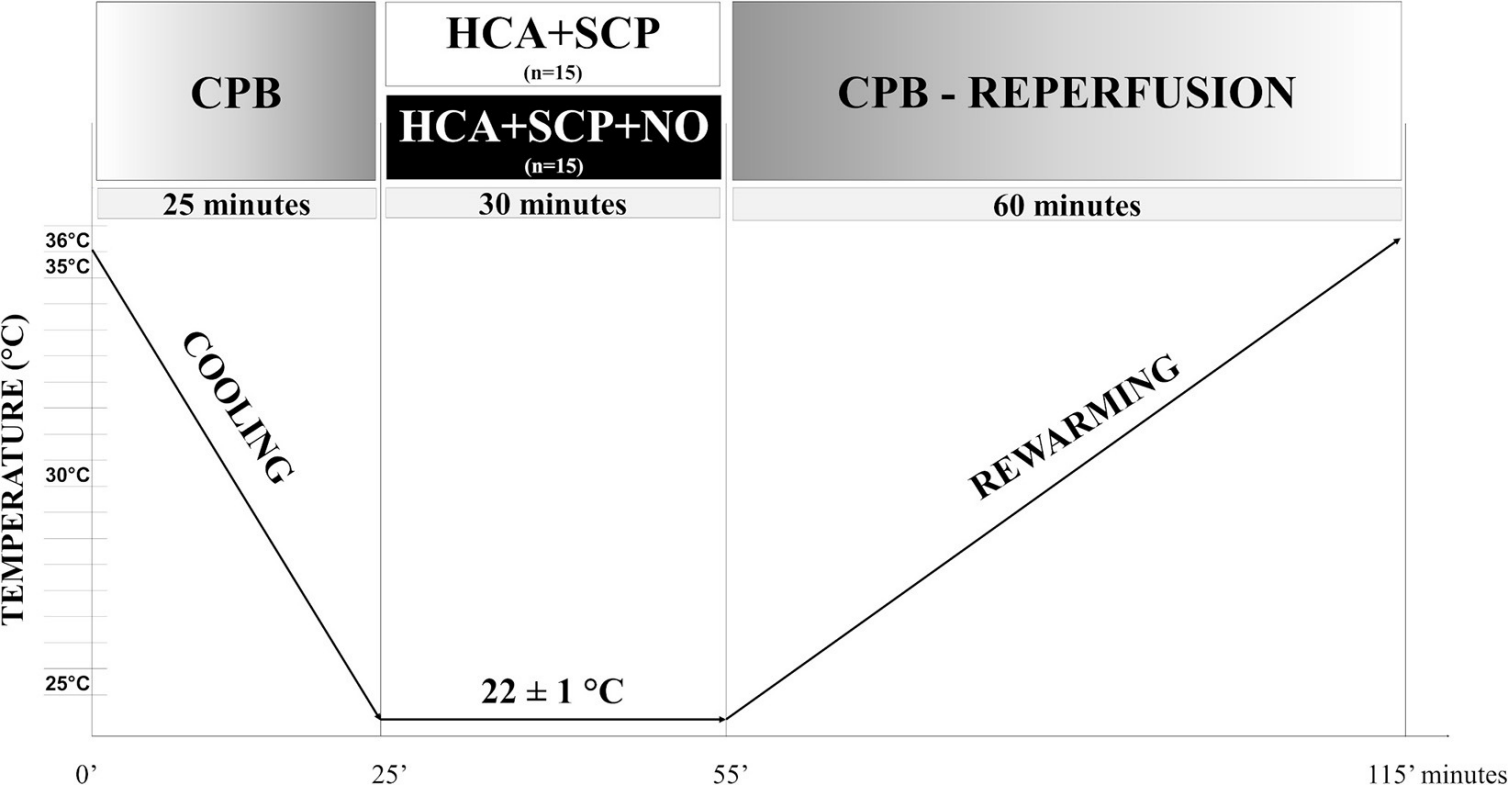
Experimental protocol. Timeline of the experimental protocol. CPB, cardiopulmonary bypass; HCA, hypothermic circulatory arrest; SCP, selective cerebral perfusion.

The dose regimen of 20 ppm of NO has been selected considering the highest dosage with a cumulative methemoglobin production minor or equal to 4% in pilot experiments. After 30 min, the aortic clamp was removed, and total CPB was re-established. After 60 min of reperfusion and rewarming to 35°C, the animals were weaned from CPB (Figure 2).

### Arterial Blood Gas Analysis

Arterial samples were collected for blood gas analysis (pH, pO_2_, pCO_2_, hematocrit, and lactate) before the procedure (T0), before HCA (T1), after HCA (T2), and at the end of the experiment (T3).

### Tissue Analysis

After injection of a lethal bolus of KCl, phosphate-buffered saline (PBS 1X, 10 ml) was injected in the cannula inserted in the upper left carotid, followed by 10 ml paraformaldehyde 4% (PFA)-4% sucrose. A posterior craniotomy was performed, and the whole brain was removed and placed in PFA 4–4% sucrose overnight. The next day brains were placed in 30% sucrose solution and kept at 4°C. About 70 slides were prepared for each sample, each with 4–5 slices (35-μm-thick) and stored at −20°C. Cryosections were incubated overnight at 4°C in the primary antibody solution. The following day, slides were left in incubation with the secondary antibody conjugated with fluorophores. Slides were kept in the dark to avoid the photobleaching of the fluorophore.

Ionized calcium binding adapter molecule 1 (Iba1) (Wako Chemicals, USA) is specific for microglia cells and macrophages that are determinants of the central nervous system's first and primary immune defense (18–20). To analyze the degree of oxidative stress and apoptosis, we evaluate the expression of 8-oxo-2′-deoxyguanosine (8oxodG) (ABCAM, UK) and caspases 3 (Cell Signaling, USA) (21–23).

After incubation with the primary and secondary antibody solution, slides were incubated with DAPI (4′,6-diamidine-2′-phenylindole dihydrochloride, molecular probes—Thermo Fisher Scientific, 1:3,000) for nucleus staining.

To detect hypoxic regions in the brain, reduced thiols were labeled (24). After perfusion with a solution containing N-ethylmaleimide (NEM) and iodoacetic acid (IAA) to block free thiols, brains were fixed with 4% PFA containing NEM and IAA. Finally, brains were dissected and kept overnight in the same solution and then processed for cryosectioning as described before. After cryosectioning, samples were washed with a solution containing tris(2-carboxyethyl) phosphine hydrochloride in PBS to reduce free disulfides. Samples were then labeled with 0.1% CPM (7-diethylamino-3-(4'-maleimidylphenyl)-4-methylcoumarin) and mounted with DABCO (1,4-diazabicyclo[2.2.2]octane).

Slides were examined with a fluorescence microscope (Eclipse Ti, Nikon, objective 20X); images obtained for each region considered were then transferred to a computer for analysis using a custom-designed ImageJ macro (US National Institutes of Health) in blind conditions.

### Statistical Analysis

Data analysis was performed using SPSS software version 21 (SPSS Inc., Chicago, Illinois), mean ± SD was calculated for all the measurements. The average percentage of cells positive for specific markers: Iba1, 8oxodG, and caspases 3 expressions were quantified in randomly selected brain regions comprising the sensory-motor cortex. The number of marker positive cells (Iba1, 8oxodG, and caspases 3) was normalized to the total number of nuclei of the regions considered (429 images were quantified across the different conditions, each image was acquired as a single image). For the hypoxic area analysis, the sensory-motor cortex was delimited, and hypoxic areas were quantified as the percentage of the mean gray level of each brain slice. The percentage of positive pixels for the hypoxic areas were normalized for the total pixel in the region considered (121 images were quantified across the different conditions, each image was acquired as a large image constituted by 6 × 20 fields).

Student *t*-test or Mann–Whitney non-parametric tests were applied to compare the immunofluorescent analysis and the blood gas values of the two groups; *p* < 0.05 was considered statistically significant.

## Results

### Arterial Blood Gas Analysis

Blood gas analysis showed an increase in lactates during HCA, followed by a gradual normalization during rewarming. The pH returned normal during rewarming after the development of acidosis in the circulatory arrest phase. No significant differences were identified between SCP and SCP-NO groups (Table 1).

**Table 1:** Blood gas parameters.

			**SCP**	**SCP-NO**	**SCP**	**SCP-NO**
	**T0 (35** **°** **C)**	**T1 (22** **°** **C)**	**T2 (22** **°** **C)**	**T2 (22** **°** **C)**	**T3 (35** **°** **C)**	**T3 (35** **°** **C)**
pH	7.40 ± 0.05	7.36 ± 0.04	7.23 ± 0.10	7.22 ± 0.12	7.38 ± 0.03	7.38 ± 0.05
pCO_2_ (mmHg)	39 ± 2.5	32 ± 3	48 ± 5.3	48 ± 3.7	32 ± 4.3	30 ± 5.8
pO_2_ (mmHg)	140 ± 10	305 ± 20	90 ± 15	87 ± 12	210 ± 33	213 ± 43
Hematocrit (%)	45 ± 2.5	47.6 ± 2.3	31 ± 2.5	32 ± 2.5	29 ± 2.8	30 ± 2.8
Lactate (mmol/l)	1.0 ± 0.2	2.5 ± 0.5	8.5 ± 2.5	8.3 ± 2.4	4.1 ± 1.6	4.6 ± 2

*SCP, selective cerebral perfusion; SCP-NO, selective cerebral perfusion plus nitric oxide. T0, baseline; T1, before hypothermic circulatory arrest (HCA); T2, after HCA; T3, end of reperfusion*.

### Inflammation

**Figure 3:**
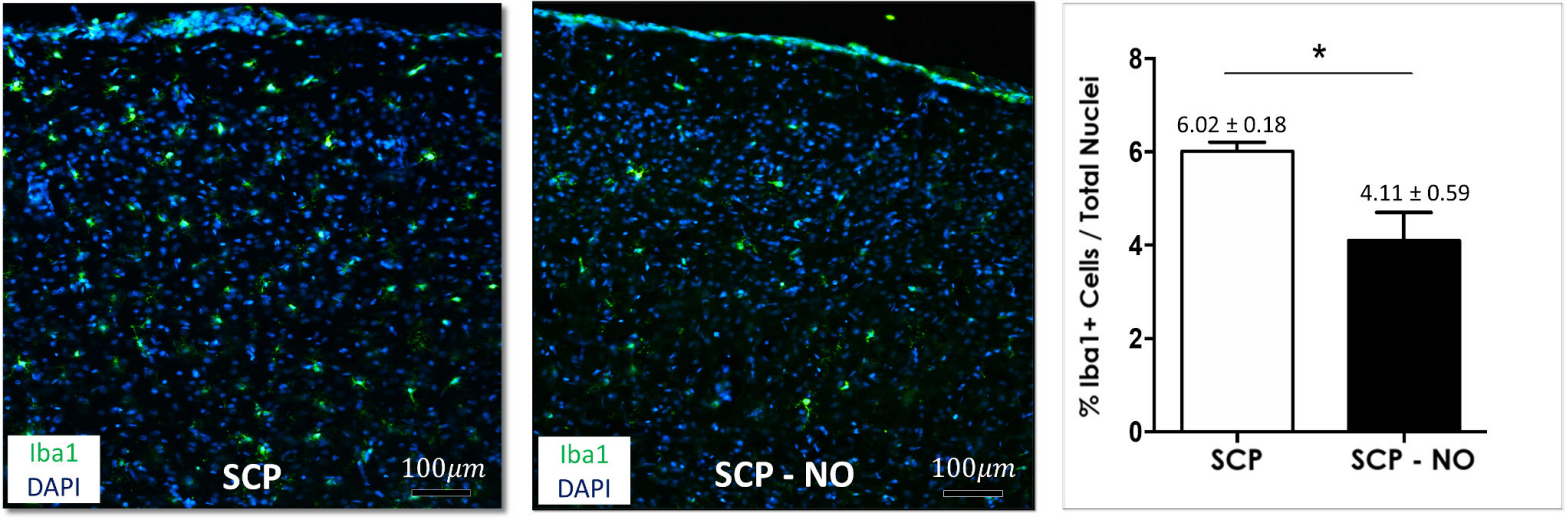
Inflammation: Iba1. Representative images of Iba1 expression at the cortical level, between the selective cerebral perfusion (SCP) group compared to selective cerebral perfusion with nitric oxide 20 ppm (SCP-NO). In the graph, blind quantification was performed to calculate the average percentage of Iba1 expression in the brain regions considered (*p < 0.05). The average percentage of Iba1 positive cells was normalized to the total number of nuclei in the regions of interest considered (170 images quantified across the different conditions) (magnification ×20). Iba1, ionized calcium-binding adapter molecule 1; DAPI, 4′,6-diamidine-2′-phenylindole dihydrochloride.

Ionized calcium binding adapter molecule 1 (Iba1) expression is specific for microglia cells and macrophages and indicates an inflammatory activation, and its amount correlates with the degree of neuroinflammation. Expression of Iba1 in different brain areas of the sensory-motor cortex in the SCP-NO group was lower compared to SCP (4.11 ± 0.59 vs. 6.02 ± 0.18%; *p* < 0.05), indicating decreased microglia activation and neuroinflammation (Figure 3).

### Oxidative Stress

**Figure 4:**
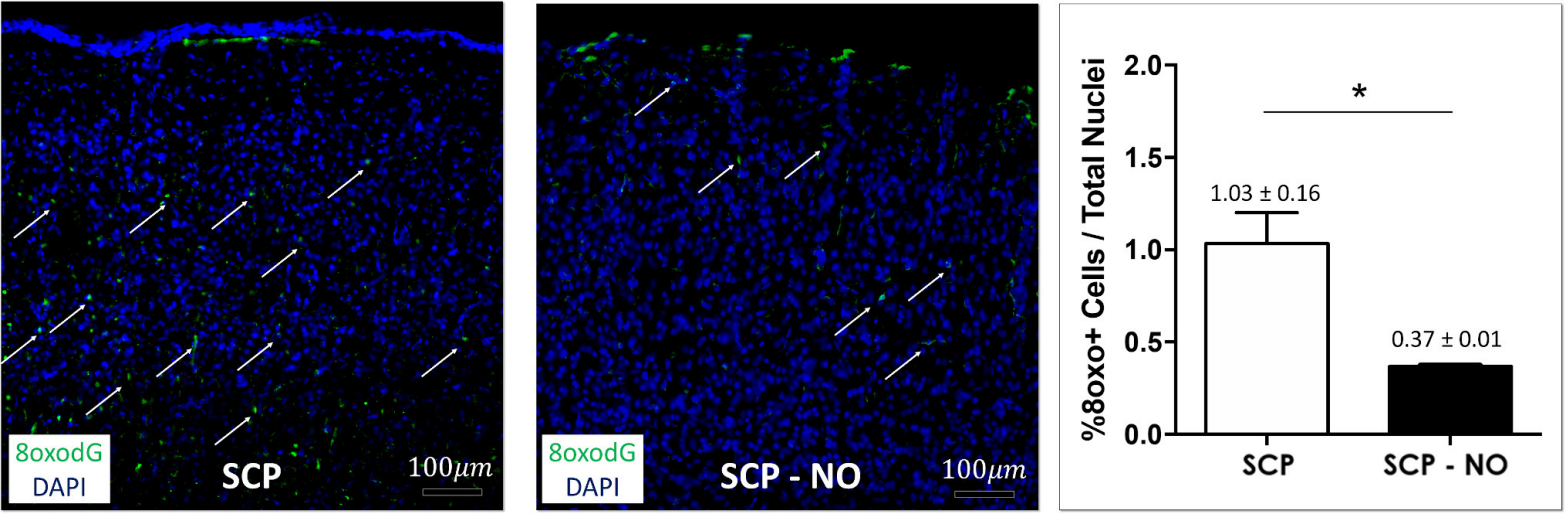
Oxidative stress: 8oxodG. Representative images of 8oxodG expression at the cortical level (arrows), in selective cerebral perfusion (SCP) group compared to selective cerebral perfusion with nitric oxide 20 ppm (SCP-NO). In the graph, blind quantification was performed to calculate the average percentage of 8oxodG expression in the brain regions considered (*p < 0.05). The average percentage of 8oxodG positive cells was normalized to the total number of nuclei in the regions of interest considered (85 images quantified across the different conditions) (magnification ×20). 8oxodG, 8-oxo-2'-deoxyguanosine. DAPI, 4′,6-diamidine-2′-phenylindole dihydrochloride.

Hypothermic circulatory arrest (HCA) and SCP contribute to ROS production and oxidative stress, which determine cell death by lipid peroxidation and DNA oxidation. The latter is associated with the conversion of the deoxyguanosine into 8-oxo-2'-deoxyguanosine (8oxoDG) that is recognized as a marker of oxidative stress. In the SCP-NO group, the 8oxoDG content was lower compared to SCP (0.37 ± 0.01 vs. 1.03 ± 0.16%; *p* < 0.05), indicating attenuated oxidative stress (Figure 4).

### Hypoxia

**Figure 5:**
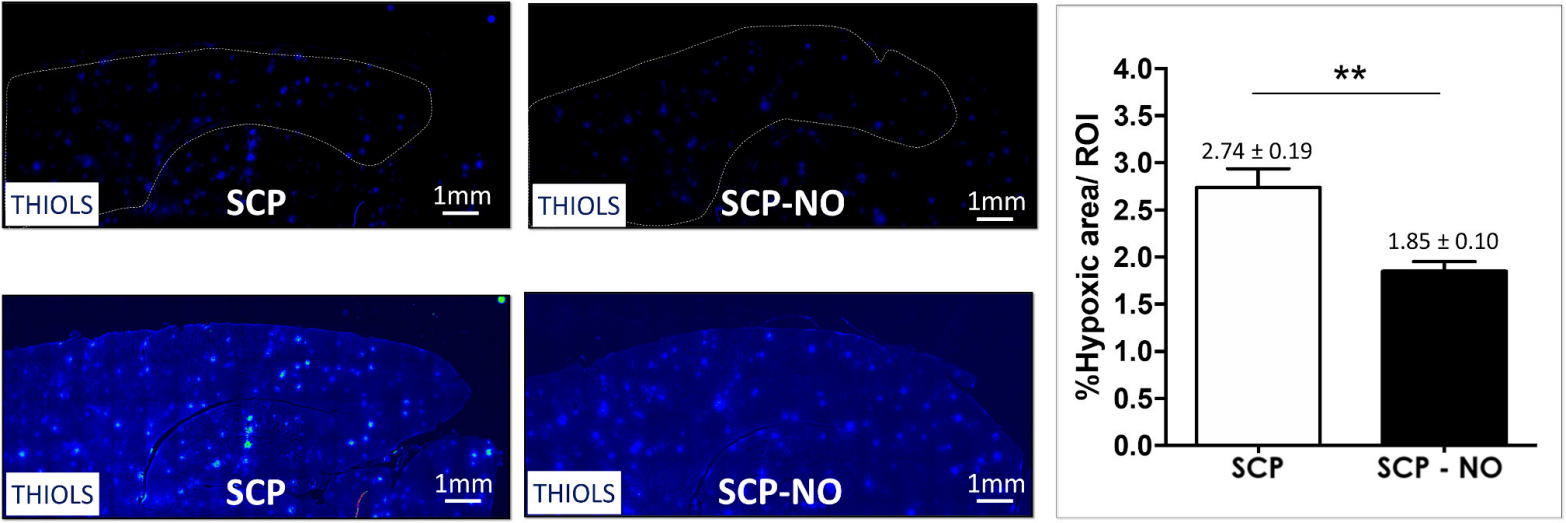
Hypoxia: thiols. Representative images of hypoxic areas at the cortical level, in selective cerebral perfusion (SCP) group compared to selective cerebral perfusion with nitric oxide 20 ppm (SCP-NO). In the graph, blind quantification was performed to calculate the average percentage of the hypoxic area extension in the brain regions considered (**p < 0.01). The average percentage of the positive pixels for the hypoxic area was normalized to the total number of pixels of the regions of interest considered (121 images quantified across the different conditions) (magnification ×20). DAPI, 4′,6-diamidine-2′-phenylindole dihydrochloride.

Thiols are a functional group of cysteine amino acids, which on the base of the oxidoreductive state could exist either as a reduced form of free thiols (-SH) or an oxidized form (-S-S-). In states of higher tissue oxygenation, thiols exist as oxidized disulfides; on the contrary, lower oxygen tissue oxygenation (hypoxia condition) leads to an increased free thiols presence. The latter indicate hypoxic brain areas, which were lower in SCP-NO compared to SCP (1.85 ± 0.10 vs. 2.74 ± 0.19%; *p* < 0.01), indicating a beneficial effect on NO in promoting the oxygen delivery counteracting impaired cerebral microcirculation (Figure 5).

### Apoptosis

**Figure 6:**
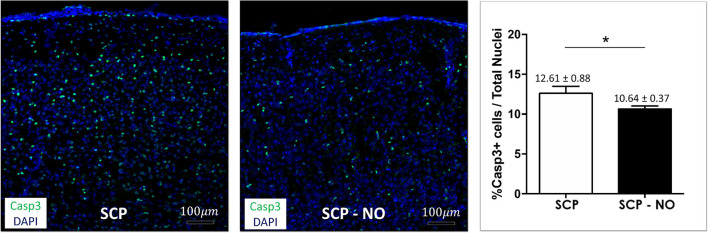
Apoptosis: caspases 3. Representative images of caspase 3 expression at the cortical level, in selective cerebral perfusion (SCP) group compared to selective cerebral perfusion with nitric oxide 20 ppm (SCP-NO). In the graph, blind quantification was performed to calculate the average percentage of caspase 3 expression in the brain regions considered (*p < 0.05). The average percentage of caspase 3 positive cells was normalized to the total number of nuclei in the regions of interest considered (174 images quantified across the different conditions) (magnification ×20). Casp3, caspases 3. DAPI, 4′,6-diamidine-2′-phenylindole dihydrochloride.

The expression of caspases 3 identifies cells where the apoptotic process is initiated. The apoptotic index (% of apoptotic cells) was lower in SCP-NO compared to SCP (10.64 ± 0.37 vs. 12.61 ± 0.88%; *p* < 0.05), demonstrating an attenuated cell death (Figure 6).

## Discussion

Hypothermic circulatory arrest (HCA) is still necessary to safely perform aortic arch surgery. Nowadays, SCP is the primary technique for brain protection in large aortic centers, while the HCA temperature has been rising progressively over the last few years (25, 26). There is a certain degree of consensus in the use of SCP and HCA; however, the temperatures and flow of perfusion vary from center to center, and there are no consensus statements, no clinical guidelines (4). Although HCA reduces brain metabolism and assures neuroprotection while permitting intervention in a clear surgical field, it can induce oxidative stress, inflammatory response, endothelial swelling, cerebral edema, and vasospasm (27). The critical role of reperfusion and simultaneous rewarming should not be underestimated since they have been demonstrated to be the main responsible for neuroinflammation and neuronal apoptosis, resulting in TND and cognitive dysfunction (28, 29).

To maximize the neurological outcome, efforts to identify possible neuroprotective agents combined with SCP should be pursued similarly to what has been done for cardioplegic solutions. The ideal pharmacological agent to add to SCP should improve perfusion distribution overcoming impaired cerebral autoregulation during hypothermia. Moreover, it should minimize ischemia-reperfusion injury responsible for neuroinflammation and apoptosis (30).

Nitric oxide (NO) administration has been studied after focal cerebral ischemia and has been demonstrated to decrease nuclear factor kappa B activation, oxidative and nitrosative stress, inflammation, and apoptosis, resulting in reduced infarct size and increased cerebral blood flow. The basal concentration is mainly due to constitutive isoforms of NO synthase (eNOS and nNOS). It is essential for regulating cerebrovascular hemodynamic and protecting endothelium integrity from inflammatory, oxidative, and procoagulant stimuli. Total suppression of eNOS activity in knockout mice makes them hypertensive and more susceptible to ischemia-reperfusion injury, with larger infarcts than in controls and severe reduction in cerebral blood flow. Conversely, nNOS gene deficiencies or nNOS inhibition decrease infarct volume and neuronal death (12).

During ischemia and hypothermia, the production of NO by constitutive NO synthase decreases. This is followed by a late burst of NO produced by inducible NO synthase (iNOS) that further increases its concentration to toxic concentrations (10). The primary sources of iNOS include microglia, astrocytes, and endothelial cells, infiltrating leukocytes, and can influence the evolution of brain damage after an ischemic injury. To the same extent microglia and macrophages express Iba1 when activated by a proinflammatory stimulus or by hypothermia (20, 31).

In this study, NO administration in SCP resulted in a reduced expression of Iba1, corresponding to lower neuroinflammation. Indeed, less activation of microglia leads to lower production of inflammatory cytokines, and prevents the expression of iNOS, thus attenuating cell damage resulting from inflammatory activation (32). This is consistent with the results obtained investigating the effects of inhaled NO on microglia after deep HCA (33).

In this study, NO administration during circulatory arrest reduced oxidative stress, as shown by the lower 8oxodG content. Cerebrovascular ischemia induces an inflammatory response and associated increased cytokine release and iNOS activity. NO, generated mainly by iNOS, reacts with superoxide and forms peroxynitrite. Peroxynitrite causes cellular necrosis with other ROS by lipid peroxidation, DNA damaging, disrupting mitochondrial respiratory chain, and ATP production (10).

Changes in eNOS expression, increased ROS production, and eNOS uncoupling are the principal reasons behind endothelial dysfunction. NADPH oxidase catalyzes the transfer of electrons from NADPH to oxygen to produce superoxide. The mitochondrial electron transport chain is another important source of superoxide in the cerebral vasculature. Oxidative stress and related cytotoxicity result in mitochondrial dysfunction, further accelerating superoxide production and neuronal death (12).

Nitric oxide (NO) administration reduces iNOS activation and eNOS uncoupling (10). In this situation, the production of peroxynitrite and superoxide is lowered, and oxidative stress on neuronal cells decreases.

In addition to the reduced neuroinflammation and oxidative stress, the reduction in hypoxic areas in animals treated with NO-enriched SCP could contribute to neuronal cell survival. Thiols groups are oxidized more uniformly, and the hypoxic area extension, detected by reduced thiols, decreases. The reduction of oxidative stress and hypoxic areas are indicative of preserved coupling between oxygen delivery and consumption. Administration of NO downregulates neuronal apoptosis by inhibiting increased phosphorylation of JNK, c-Jun, and Bcl-2. Furthermore, NO can nytrosilate caspases 3 directly and prevent the activation of apoptosis (12). These effects, along with an increase in cerebral blood flow, reduce brain damage after the ischemia-reperfusion event (21).

All these elements with an attenuated inflammatory milieu explain the reduced apoptosis index demonstrated in brains perfused by SCP added with NO. Indeed, NO significantly decreased caspases 3 positive cells, demonstrating a positive effect of NO administration on cell survival.

A possible limitation of this study is that it was performed on a small animal model constituted of healthy rats and with data collected in an early phase (1 h) after hypothermic circulatory arrest.

However, the protocol is clinically relevant and assured continuous SCP during 30-min HCA. The two groups studied were equivalent, and there were no vascular alterations; the consequence was diffuse and non-focal brain damage. The latter was measured by immunofluorescence analysis, and it is not possible to diagnose through current clinical techniques. Clinical manifestations can range from a slow awakening after anesthesia to delirium and psychomotor agitation resulting in TND. This study aimed to address this type of neurological damage, which negatively impacts neurologic outcomes and prognosis after aortic arch surgery.

The results of this study could inspire prospective randomized clinical studies to investigate NO application as a neuroprotective agent during SCP and HCA.

## Conclusions

Nitric oxide (NO) administration in the oxygenator during SCP and HCA may improve neuroprotection decreasing neuroinflammation, optimize oxygen delivery by reducing oxidative stress and hypoxic areas finally decrease apoptosis.

Further experiments are needed to investigate the effects on neurological outcomes.

## Data Availability Statement

The raw data supporting the conclusions of this article will be made available by the authors, without undue reservation.

## Ethics Statement

The animal study was reviewed and approved by the Scientific Committee for Experimental Research of the University of Verona and approved by the National Ministry of Health (Ministerial Authorization Number 568/2020-PR, Protocol Number 56DC9.54).

## Author Contributions

DL: conceptualization, methodology, investigation, and writing-original draft. AR: conceptualization project administration, writing-review, and editing. GF and GL: funding acquisition and supervision. LG: resources acquisition (nitric oxide). SM and MT: methodology and investigation (cardiopulmonary bypass technician). ID, LM, and SD: formal analysis (immunofluorescence). AM and RM: methodology, investigation, and experiment execution. All authors contributed to the article and approved the submitted version.

## Funding

Experiments and analyses were financially sustained by the internal funds of Prof. Faggian, Prof. Luciani, and Prof. Gottin with the University of Verona.

## Conflict of Interest

The authors declare that the research was conducted in the absence of any commercial or financial relationships that could be construed as a potential conflict of interest.

## Publisher's Note

All claims expressed in this article are solely those of the authors and do not necessarily represent those of their affiliated organizations, or those of the publisher, the editors and the reviewers. Any product that may be evaluated in this article, or claim that may be made by its manufacturer, is not guaranteed or endorsed by the publisher.

## Abbreviations

CPB: Cardiopulmonary bypass
DAPI: 4′, 6-Diamidine-2′-phenylindole dihydrochloride
HCA: Hypothermic circulatory arrest
Iba1: Allograft inflammatory factor 1 or ionized calcium-binding adapter molecule 1
NO: Nitric oxide
NOS: Nitric oxide synthase
PND: Permanent neurological dysfunction
ROS: Reactive oxygen species
SCP: Selective cerebral perfusion
TND: Temporary neurological dysfunction.
